# Size Matters: An Evaluation of the Molecular Basis of Ontogenetic Modifications in the Composition of *Bothrops jararacussu* Snake Venom

**DOI:** 10.3390/toxins12120791

**Published:** 2020-12-11

**Authors:** Luciana A. Freitas-de-Sousa, Pedro G. Nachtigall, José A. Portes-Junior, Matthew L. Holding, Gunnar S. Nystrom, Schyler A. Ellsworth, Noranathan C. Guimarães, Emilly Tioyama, Flora Ortiz, Bruno R. Silva, Tobias S. Kunz, Inácio L. M. Junqueira-de-Azevedo, Felipe G. Grazziotin, Darin R. Rokyta, Ana M. Moura-da-Silva

**Affiliations:** 1 Programa de Pós-Graduação em Ciências-Toxinologia, Laboratório de Imunopatologia, Instituto Butantan, 05503-900 São Paulo, SP, Brazil; noranathan.guimaraes@butantan.gov.br (N.C.G.); emilly.tioyama@butantan.gov.br (E.T.); 2 Laboratório Especial de Toxinologia Aplicada, Instituto Butantan, 05503-900 São Paulo, SP, Brazil; pedronachtigall@gmail.com (P.G.N.); inacio.azevedo@butantan.gov.br (I.L.M.J.-d.-A.); 3 Laboratório de Coleções Zoológicas, Instituto Butantan, 05503-900 São Paulo, SP, Brazil; portes.junior@butantan.gov.br (J.A.P.-J.); flora_bio@hotmail.com (F.O.); brunorochaherpeto@gmail.com (B.R.S.); tskunz@gmail.com (T.S.K.); felipe.grazziotin@butantan.gov.br (F.G.G.); 4 Department of Biological Science, Florida State University, Tallahassee, FL 32306, USA; matthewholding28@gmail.com (M.L.H.); gnystrom@bio.fsu.edu (G.S.N.); sellsworth@bio.fsu.edu (S.A.E.); drokyta@bio.fsu.edu (D.R.R.); 5 Instituto de Pesquisa Clínica Carlos Borborema, Fundação de Medicina Tropical Dr. Heitor Vieira Dourado, 69040-000 Manaus, AM, Brazil

**Keywords:** *Bothrops jararacussu*, ontogenetic variability, transcriptome, proteome, metalloproteinases, phospholipases A_2_

## Abstract

Ontogenetic changes in venom composition have been described in *Bothrops* snakes, but only a few studies have attempted to identify the targeted paralogues or the molecular mechanisms involved in modifications of gene expression during ontogeny. In this study, we decoded *B. jararacussu* venom gland transcripts from six specimens of varying sizes and analyzed the variability in the composition of independent venom proteomes from 19 individuals. We identified 125 distinct putative toxin transcripts, and of these, 73 were detected in venom proteomes and only 10 were involved in the ontogenetic changes. Ontogenetic variability was linearly related to snake size and did not correspond to the maturation of the reproductive stage. Changes in the transcriptome were highly predictive of changes in the venom proteome. The basic myotoxic phospholipases A_2_ (PLA_2_s) were the most abundant components in larger snakes, while in venoms from smaller snakes, PIII-class SVMPs were the major components. The snake venom metalloproteinases (SVMPs) identified corresponded to novel sequences and conferred higher pro-coagulant and hemorrhagic functions to the venom of small snakes. The mechanisms modulating venom variability are predominantly related to transcriptional events and may consist of an advantage of higher hematotoxicity and more efficient predatory function in the venom from small snakes.

## 1. Introduction

Advanced snakes (clade Caenophidia) have acquired the ability to produce, secrete, and inject venoms. The evolution of lethal toxins and the appearance of the sophisticated injection system provided snakes a great advantage in the evolutionary process to subdue prey [1]. Snake venoms are mainly composed of proteic toxins, the genes of which have been recruited early during Caenophidia diversification. These ancestor genes underwent duplications, and the copies recruited for venom were diversified through several genetic mechanisms resulting in neofunctionalization [1,2,3,4]. The differential expression of those distinct paralogues allows for a wide diversity of snake venom phenotypes and an astonishing variability observed across multiple taxonomic and intraspecific levels. The high degree of variability in venom composition is implicated as a key factor in the occupation of different ecological niches by the advanced snakes [1,5].

Among the Caenophidia, the family Viperidae evolved one of the most complex arsenals of venom toxins. The major protein families present in viper venoms are snake venom metalloproteinases (SVMPs), snake venom serine proteinases (SVSPs), phospholipases A_2_ (PLA_2_), and C-type lectins (CTLs) [6]. Each of these toxin families includes a large number of isoforms with variable expression levels [7]. In vipers, venom variability can be observed between different species [8,9] and intraspecifically, it is represented by variation associated with ontogeny [10,11,12], sex [13], geographic distribution [14,15,16], or local adaptation to different environmental conditions [7,17].

The presence of such a high variability in venom composition makes many viper species ideal models for studying venom evolution. *Bothrops* is one of the most diverse genera of Viperidae [18]. This group is widely distributed in Central and South America and on the Lesser Antilles islands of Saint Lucia and Martinique [19]. The generalist habit of most species of *Bothrops* is one of the factors responsible for their high abundance and the wide diversity of habitats they occupy [20]. *Bothrops jararacussu* is among the largest species of *Bothrops* and can reach up to 1.8 m in total length. The species is an endemic dweller of the Brazilian Atlantic Forest [21] and presents the largest known sexual size dimorphism for the genus [22]. Females are much larger than males, and they have larger heads that help produce higher venom quantities, allowing the capture and ingestion of larger prey [23]. Adults feed predominantly on mammals, while juveniles feed mainly on ectothermic prey, such as amphibians [24].

The venom of *B. jararacussu* is also distinct in the genus. In general, venoms of *Bothrops* snakes contain a high abundance of SVMPs, while *B. jararacussu* venom contains a higher abundance of PLA_2_s [25]. Recently, differences in the venoms of juvenile and adult *B. jararacussu* specimens of captive snakes have been identified, and ontogenetic variability has been reported in groups fed with a fixed diet [26]. However, one relevant shortcoming of those previous studies is that the variability in snake venom composition was evaluated mostly on the presence and quantification of the protein families composing the toxic arsenal. It is currently accepted that different isoforms from the same protein family may contribute differently to venom toxicity. A clear example of that is the abundance of a basic PLA_2_ analogue in venoms of *Bothrops* snakes in which the aspartic acid in position 49 is replaced by a lysin. Those analogues, recognized as K49-PLA_2_, present very weak catalytic activity since the aspartic acid in such a position is important for catalysis [27]. On the other hand, K49-PLA_2_s conserve the major toxic function of this toxin family and are responsible for the high myotoxic activity present in some venoms of *Bothrops* snakes, particularly of *B. jararacussu* [27]. Concerning SVMPs, the isoforms isolated from the venom of *Bothrops neuwiedi* induce hemostatic damages by distinct mechanisms and different selectivity to avian or mammalian prey [28].

Free-label proteomics quantification based on a species genome or transcriptome is a powerful tool for characterizing and quantifying isoforms in venom toxin groups [29] and allows for a better understanding of the participation of independent isoforms in venom adaptive strategies [7,30,31]. Thus, transcriptome-based free-label proteomics enables the characterization of isoforms present in venoms from snakes collected in their natural environment and subjected to their natural diet. Using this strategy, we aimed to understand if ontogenetic variability also occurs in the venom of *B. jararacussu* under natural conditions and whether a major variation is restricted to specific toxin paralogues. Furthermore, we investigate the molecular mechanisms involved in ontogenetic variation in *B. jararacussu* and discuss whether variation targeting isoforms with particular functions reflect adaptive advantages for the species. 

## 2. Results

### 2.1. Intraspecific Variability of B. jararacussu Venom Composition

The variability in venom composition of *B. jararacussu* snakes was analyzed in this study using a group of nineteen snakes recently collected in São Paulo, Santa Catarina, and Rio de Janeiro states of Brazil (Supplementary Figure S1), including ten females and nine males with snout-vent lengths (SVL) ranging from 257 to 1230 mm (Supplementary Table S1). According to histological analyses of reproductive organs (data not shown), eight individuals were considered adults, and eleven were juveniles. For transcriptomic analysis, we sequenced the venom glands of six individuals of different sizes with Illumina technology, resulting in more than 14 million reads for each sample. The merging of the paired reads based on their 3′ overlaps was over 80% for all samples (Supplementary Table S2). Then, these sequences were assembled and annotated for identification and quantification. Toxin-coding sequences presenting 98% identity to each other were clustered to generate a final master set containing 125 different toxin isoforms of all major toxin groups (Supplementary Table S3).

The most common families of toxins from the *Bothrops* genus were identified: BPP (Bradykinin Potentiating Peptide), CTL (C-Type Lectin), PLA_2_ (Phospholipase A2), SVMP classes PI, PII and PIII (Snake Venom Metalloproteinase), SVSP (Snake Venom Serine Protease), VEGF (Vascular Endothelial Growth Factor), LAAO (L-amino Acid Oxidase), CRISP (Cysteine-rich secretory protein), HYAL (Hyaluronidase), KUN (Kunitz-type protease inhibitor), VNGF (Venom Nerve growth factor), SVNUC (Snake Venom Nucleotidase), PDE (Venom Phosphodiesterase), WAP (Waprin-Whey Acidic Protein-type), PLB (Phospholipase B), PLA_2_ inhibitor, Cystatin, and GLUT (Venom glutaminyl cyclase). The greatest transcript diversity was detected among the CTL, SVSP, SVMP, and PLA_2_ families, in which there were 26, 24, 31, and 10 isoforms, respectively. The other families of toxins presented a smaller number of distinct transcripts. The annotation of these sequences is detailed in Supplementary Table S3.

**Figure 1:**
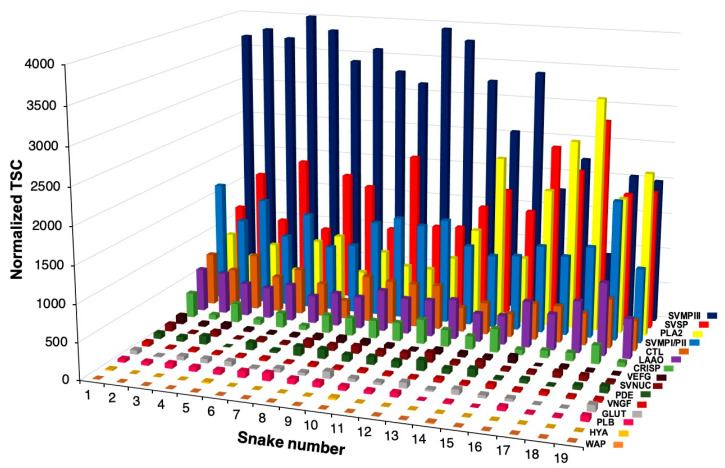
Distribution of the toxin families in the venom of 19 specimens of *Bothrops jararacussu* identified from proteome analysis. Relative expression is indicated by the normalized Total Spectrum Count (TSC) in each snake. CTL (C-Type Lectin), PLA_2_ (Phospholipase A_2_), SVMP classes PI, PII, and PIII (Snake Venom Metalloproteinase), SVSP (Snake Venom Serine Protease), VEGF (Vascular Endothelial Growth Factor), LAAO (L-amino Acid Oxidase), CRISP (Cysteine-rich secretory protein), HYAL (Hyaluronidase), VNGF (Venom Nerve Growth Factor), SVNUC (Snake Venom Nucleotidase), PDE (Venom Phosphodiesterase), PLB (Phospholipase B) and GLUT (Snake Venom Glutaminyl Cyclase). The higher the snake number, the greater the size of the snake.

The translated sequences of the master set generated from the transcriptomic analysis, comprising the 125 distinct isoforms, were used as a database to identify peptides detected by LC-MS/MS from individual venom samples. The expression levels of each toxin group in venom proteomes were evaluated by normalized Total Spectral Counting (TSC) and are shown in Figure 1 and detailed in Supplementary Table S4. In general, the most abundant venom components were SVMPs (12.97–59%), PLA_2_s (4.77–37.3%), SVSPs (11.18–33%), LAAOs (4.44–11.56%), CTLs (2.97–8.98%), and CRISPs (0.7–3.98%). SVNUCs, PDEs, VNGFs, GLUTs, and PLBs presented levels below 2% in all individuals, HYAL was detected (<4%) in only 4 specimens, and WAP was detected only in BJSU14 (0.1%). CYS, KUN, and BPP groups were not detected in the venoms. Interestingly, PI-class SVMPs were detected only in the venoms of five specimens. However, in the mature form, these classes of SVMPs may have originated by the post-translational processing of either PI or PII-class precursors [32]. Taking this into account, we gathered the proteomic data generated by PI- and PII-class precursors in one group representing the PI/PII-class SVMPs.

**Figure 2:**
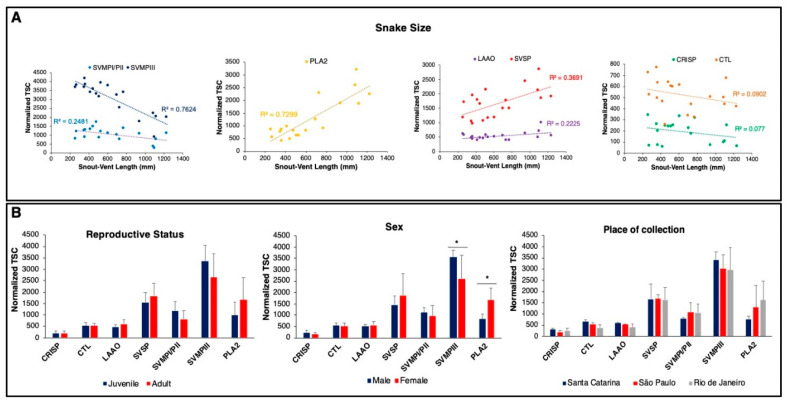
Correlation of the main toxin families of *Bothrops jararacussu* venom with: (**A**) the individuals’ snout-vent length (SVL), (**B**) reproductive status, sex, and place of collection. Relative expression is indicated by the normalized Total Spectrum Count (TSC) in each snake. CTL (C-Type Lectin), PLA_2_ (Phospholipase A_2_), SVMP classes PI, PII and PIII (Snake Venom Metalloproteinase), SVSP (Snake Venom Serine Protease), LAAO (L-amino Acid Oxidase), CRISP (Cysteine-rich secretory protein). * *p* < 0.05.

The predominance of the major toxin groups was consistent across the venoms of all specimens. However, the distribution of each toxin group was not uniform amongst the different venoms: PIII-class SVMPs were abundant in snakes BJSU01–BJSU12 and BJSU14, while the distribution of PLA_2_s predominated in the venoms of snakes BJSU13 and BJSU15-BJSU19. According to the present data, we noticed a tendency for a higher abundance of PLA_2_s in larger individuals, whereas the PIII-class SVMPs are the most abundant toxins in smaller individuals. Taking this into account, our next step was to evaluate if there was any correlation between the expression levels of the most abundant toxin families in *B. jararacussu* venoms to the snake size, reproductive status, sex, and locality of collection individual snakes (Figure 2).

Figure 2A shows the correlation between the snake size and the abundance of the major toxin groups (normalized TSC). SVMPs are the major component in most individuals; however, there is a marked decrease in PIII-class SVMPs as the snakes increased in size (R^2^ = 0.76/*p* = 0.00003/slope b = −2.504), denoting a significant inverse correlation between both parameters. The PI/PII SVMPs showed a weak correlation with the size of the individuals (R^2^ = 0.24/*p* = 0.029/slope b = −0.576). On the other hand, the abundance of venom PLA_2_s was positively correlated to the size of individuals (R^2^ = 0.72/*p* = 0.000001/slope b = 2.244). The other most abundant components showed a more even distribution across all venoms. SVSPs and LAAOs showed a low positive correlation to snake size (R^2^ = 0.36/*p* = 0.006/slope b = 0.983 and R^2^ = 0.22/*p* = 0.0052/slope b = 0.211, respectively) and the abundance of CTLs and CRISPs did not correlate according to snake size. 

The predominance of PIII-class SVMPs in the venom of juvenile individuals has already been shown in venoms of *Bothrops* snakes and correlated to the ontogenetic stage of the snakes [11,14]. However, the definition of sexually mature individuals is usually assumed based on body size and not on the actual observation of the reproductive organs. Thus, the next step was to compare the abundance of each toxin group in venoms of juvenile/adult or male/female snakes defined by histological analyses of the reproductive organs of each snake. Regardless of the strong correlation observed between the total amount of PIII-class SVMPs and PLA_2_ with the snake size (Figure 2A), the amount of these toxins was not statistically different in the snakes grouped according to the reproductive status (Figure 2B), suggesting that venom variability was not due to the sexual maturation of the snakes. Comparing venom composition in snake sexes, levels of PIII-class SVMPs predominated in males, while PLA_2_ levels were higher in females (Figure 2B). It is already known that *B. jararacussu* snakes have great sexual size dimorphism, in which males are much smaller than females [22]. Thus, the observed difference related to snake sex may be because the largest individuals are females. However, it is important to note that the largest male included in the study (BJSU014) contains predominantly PIII-class SVMP in its venom, resembling the venoms of small specimens. Concerning the locality of collection, the venom of *B. jararacussu* from specimens collected in three different Brazilian states (Supplementary Figure S1) showed no significant differences in the quantity of the main toxin families (Figure 2B). To clarify whether the differences found between males and females in SVMPs and PLA_2_s abundances was due to sex or the larger size of collected females, we performed an analysis of multiple linear regression that includes both size, sex, locality of collection, and reproductive status as independent variables. The result obtained was the same when we performed Pearson and Spearman correlation analyses, and only the snake size showed a significant correlation with the expression of SVMPs (*p* < 0.0001) and PLA_2_ (*p* = 0.0026) (data not shown).

### 2.2. Specific Isoforms Characterize B. jararacussu Venom Variability

Our next step was to do a more in-depth analysis of the isoforms comprised within these toxin families in the venom of *B. jararacussu* from different sizes. For the estimate of isoform levels, we used normalized Exclusive Unique Spectral Counting (EUSC) to avoid redundancy due to the similarity between the isoforms present in each family of proteins (Supplementary Table S4). In addition, to estimate the functional relevance of the isoforms, we aligned our sequences with selected similar PLA_2_s and PIII-class SVMPs isolated from other venoms with functions well-established by previous studies using experimental approaches.

**Figure 3:**
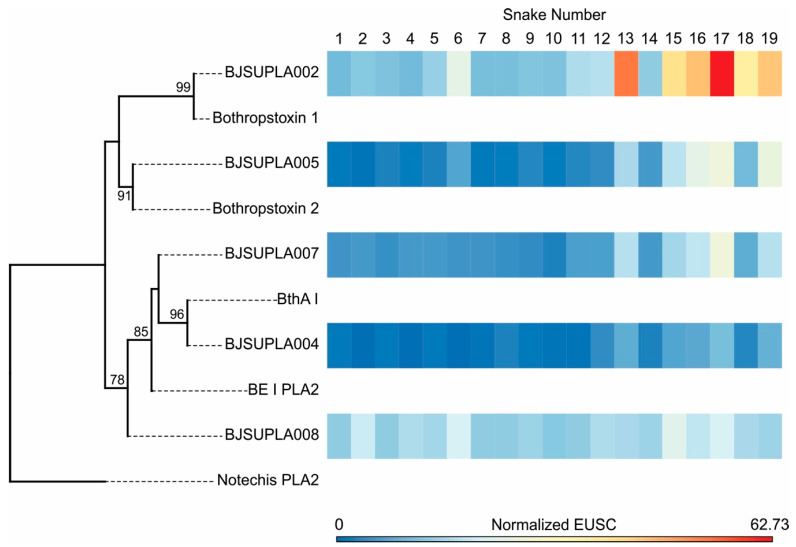
Distribution of PLA_2_ isoforms present in the venoms of 19 individuals from *B. jararacussu* and functional inferences. Mature deduced protein sequences of PLA_2_ were aligned together with sequences of PLA_2_s isolated from other venoms with functions well-established by previous studies using experimental approaches, as follows: a non-toxic phospholipase A2 from the venom of *Notechis scutatus scutatus*; Bothropstoxin-1 (Q90249.3), Bothropstoxin-2 (P45881) and BthAI (Q8AXY1.1), *B. jararacussu* venom; acidic PLA_2_ (Q2HZ28.1), *B. erythromelas* venom. *Notechis* PLA_2_ (P08873.1) sequence was used to root the tree. The maximum likelihood phylogenetic tree was generated using RaxML (v8.2.12; Stamatakis 2014). The heatmap on the right indicates the number of normalized Exclusive Unique Spectrum Counts (EUSC) of MS/MS in each venom sample, which are as indicated at the top of the figure. Bootstrap values are described only when greater than 75.

Regarding PLA_2_s, 10 isoforms were identified in the transcriptome, of which only five were detected in the venom proteome. The isoforms BJSUPLA002 and BJSUPLA008 were the most abundant in all venom samples. EUSC levels of BJSUPLA008 were similar in snakes of different sizes. In opposition, EUSC levels of BJSUPLA002 and BJSUPLA005 were approximately four times higher in the largest individuals (SVL > 600 mm). BJSUPLA004 and BJSUPLA007 were not abundant in the venoms but also presented higher levels (2–3 times) in bigger snakes (Figure 3; Supplementary Table S4). The prevalent sequences in bigger snakes, BJSUPLA002 and BJSUPLA005, clustered together with basic myotoxic PLA_2_ isoforms. BJSUPLA002 corresponds to the previously isolated *B. jararacussu* BthTX-1 (100% identity), which is a myotoxic K49-PLA_2_ analogue devoid of enzymatic activity in vitro [33], whereas BJSUPLA005 presented 100% identity with BthTX-2, a myotoxic D49-PLA_2_ isolated from the same venom, but displaying phospholipase catalytic activity [34]. Contrasting to that, sequences clustered with the acidic PLA_2_s were more evenly distributed among the venoms. BJSUPLA004 presented 100% identity with BthA-I-PLA_2_, which is a catalytically active D49-PLA_2_ previously isolated from *B. jararacussu* venom that showed anticoagulant activity upon human plasma and inhibited phospholipid-dependent platelet aggregation induced by collagen or ADP [35]. BJSUPLA007 presented 94% identity with BE-I-PLA_2_, which is a PLA_2_ isolated from *Bothrops erythromelas* venom displaying catalytic activity and the potent activity of platelet aggregation inhibitor [36]. BJSUPLA008 was the most distinct isoform detected in the venom, with low identity with other PLA_2_s described in the literature. Thus, the great variability in PLA_2_ content according to *B. jararacussu* body size is associated with the basic myotoxic forms rather than with the acidic forms.

**Figure 4:**
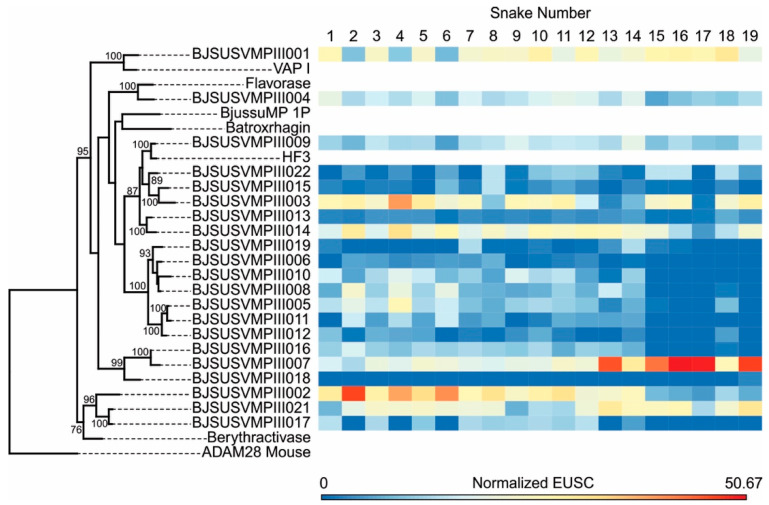
Distribution of SVMPIII isoforms present in the venoms of 19 individuals from *B. jararacussu* and functional inferences. Mature deduced protein sequences of SVMPIII were aligned together with sequences of SVMPs isolated from other venoms with functions well-established by previous studies using experimental approaches, as follows: BjussuMP-1P (Q1PHZ4; from *B. jararacussu* venom; Batroxrhagin, *Bothrops atrox* venom (ALB00542.1); hemorrhagic factor 3 (HF3), *B. jararaca* venom (Q98UF9.3); vascular apoptosis-inducing protein 1 (VAP-1), *Crotalus atrox* venom (Q9DGB9.1); berythractivase, *B. erythromelas* venom (Q8UVG0.1); VaF1, *Vipera ammodytes ammodytes* venom (AJC52543). The disintegrin and metalloproteinase domains of *Mus musculus* ADAM28 (NP034212.1) was used to root the tree. The maximum likelihood phylogenetic tree was generated using RaxML (v8.2.12; Stamatakis 2014). The heatmap on the right indicates the number of normalized Exclusive Unique Spectrum Counts (EUSC) of MS/MS in each venom sample, which are as indicated at the top of the figure. Bootstrap values are described only when greater than 75.

Of all the isoforms of PIII-class SVMPs identified in the transcriptome, only one was not detected in the proteome (BJSUSVMPIII020). The most abundant isoforms were BJSUSVMPIII001, 002, 003, 007, 014, and 021, followed by BJSUSVMPIII004, 005, 008, 009, 010, 011, 016, and 017 isoforms, with slightly lower expression (Figure 4; Supplementary Table S4). Isoforms 001, 009, and 021 presented an even distribution concerning snake sizes. However, in an opposite pattern than PLA_2_s, most PIII-class SVMPs predominated in the venoms of small snakes, particularly the isoform SVMPIII002, which was approximately four times more abundant in venoms of small snakes (SVL < 942 mm). Isoforms BJSUSVMPIII005, 008, 010, 011, 016, and 017 were almost absent in venoms of larger snakes (SVL > 770 mm). Interestingly, isoform BJSUSVMPIII007 was one of the most expressed isoforms in all venoms, but it was two to three times more abundant in venoms of larger individuals (SVL > 942 mm). Regarding the identity of these isoforms with toxins with function already characterized in experimental models, most isoforms were distinct from the ones isolated from other venoms, including the BJSUSVMPIII007, which is abundant in venoms of larger snakes. We observed some similarities between SVMPIII009 and the hemorrhagic toxin, HF3 [37]. BJSUSVMPIII004 was grouped with the Flavorase, which is a non-hemorrhagic metalloproteinase [38], and BJSUSVMPIII001 was similar to VAP-1, which induces apoptosis in vascular endothelial cells [39]. Isoforms BJSUSVMPIII002, 017, and 021 were located in the same cluster with Berythractivase, a non-hemorrhagic SVMP that is able to activate prothrombin without the presence of calcium [40]; however, it has lower similarity. It is important to note that BjussuMP-1P, the only PIII-class SVMP already isolated from *B. jararacussu* venom, with characterized biological activities [41], was not detected in our transcriptomes.

In summary, among PLA_2_s and SVMPs, we highlight BJSUSVMPIII002 and BJSUSVMPIII010 as the most highly expressed in small snakes, BJSUPLA002, BJSUPLA005, and BJSUSVMPIII007 as the most highly expressed in larger snakes, and BJSUPLA008, BJSUSVMPIII009, and BJSUSVMPIII021 evenly distributed expression despite the snake size.

### 2.3. Insights on the Mechanisms Regulating B. jararacussu Venom Variability

Considering the size-related variability of the major toxin families observed in the venom proteomes, we decided to evaluate if such differences in venom composition could be related to transcriptional events. Thus, we analyzed the transcripts and their abundance in the transcriptomes from six individuals of different sizes. As observed in proteomes, the most abundant transcripts in venom glands of the six specimens of *B. jararacussu* belonged to the PLA_2_ and SVMP toxin groups (Supplementary Figure S2; Supplementary Table S5). As observed in the proteomes, in transcripts of smaller *B. jararacussu* snakes (BJSU02, 04 and 06), PIII-class SVMPs were the most abundant transcripts (46–61%) with lower amounts of PLA_2_s (16–22%). These results contrasted with the transcripts of larger snakes (BJSU13, 15, and 17), which presented a higher expression of PLA_2_ and lower expression of PIII-class SVMP transcripts. Particularly, the snake BJSU17 (SVL—1099 mm) presented 70% of transcripts related to PLA_2_s and only about 3% related to SVMPs. On the other hand, the results of specimens BJSU13 and BJSU15, which had intermediate sizes (SVL—732 and 942 mm, respectively), demonstrated an intermediate expression pattern with similar expression levels between SVMPs and PLA_2_s. In these individuals, SVMP was the most abundant toxin family (32% and 48%); however, class PIII SVMPs showed lower abundance, and there was a proportional increase in PII-class transcripts. In addition, PLA_2_ levels were also intermediate, corresponding to 31% and 40% for BJSU13 and BJSU15, respectively. Regarding the other predominant components, levels of CTLs and SVSPs ranged from 4 to 20% and transcripts of BPPs, SVNUC, CRISP, PDE, CYS, VNGF, GLUT, KUN, WAP, and PLB were less abundant in the venom glands.

Analyzing the four more expressed toxin families, we detected a similar pattern as observed in the proteomes, with a statistically significant inverse correlation in the amount of PIII-class SVMP transcripts (R² = 0.7081/*p* =0.0057/slope b = −83.28) and a positive correlation, but not statistically significant, in the amount of PLA_2_ transcripts (R² = 0.4088/*p* = 0.17/slope b = 165.23). The other two most expressed toxin groups, SVSPs and CTLs, did not show variations in transcript abundance according to the increase of SVL.

**Figure 5:**
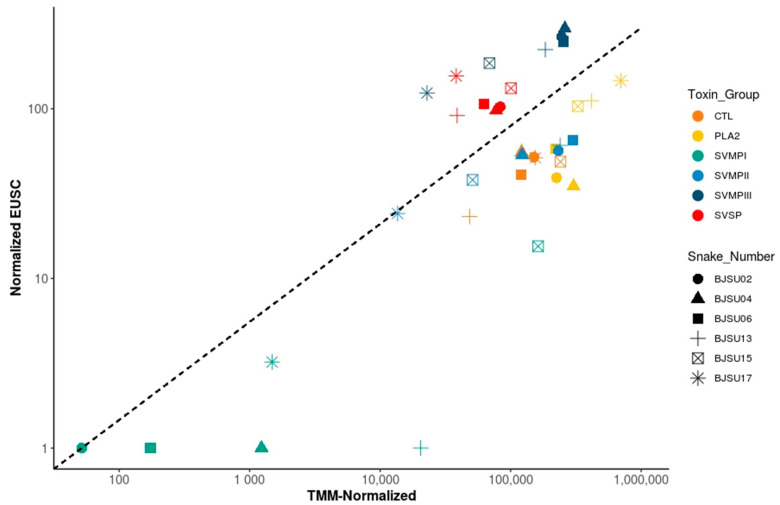
Comparisons of gene transcription (TMM normalized) rates in the venom gland and protein abundance (normalized EUSC—Exclusive Unique Spectrum Count) in the venom of individual *B. jararacussu* snakes. Each spot represents detected transcription and translation levels for a specific toxin family from one specimen. Dotted lines indicate a hypothetical correspondence between transcript and protein abundances.

The correlation between the expression levels of transcripts encoded by each gene family and their cumulative proteomic abundance in each snake specimen is shown in Figure 5. In general, the level of each toxin group in the venom correlated well with the transcriptional activity of the genes. PIII-class SVMPs and SVSPs showed a slightly higher abundance in the proteome while PLA_2_s, CTLs, and PII-class SVMPs were more abundant in the transcriptomes. This evidence suggests that most of the variability in the venom composition of *B. jararacussu* snakes is related to the transcriptional steps.

**Figure 6:**
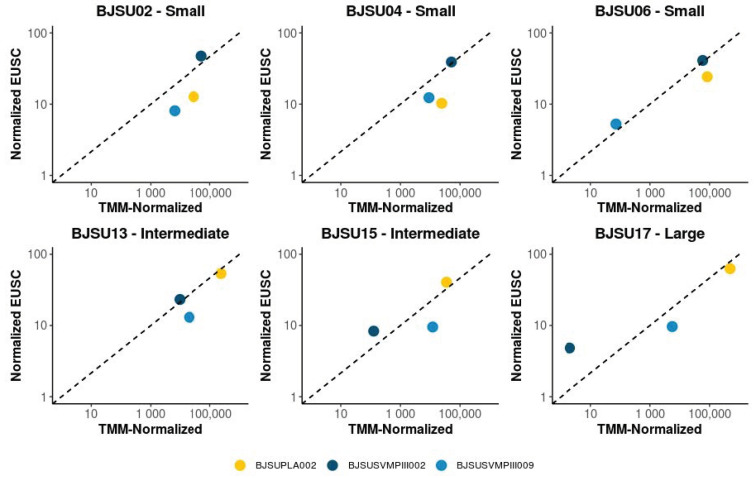
Comparison between levels of transcripts (TMM normalized) and protein abundance (normalized EUSC—Exclusive Unique Spectrum Count) of the isoforms PLA002, SVMPIII002, and SVMPIII009 in the venoms of *B. jararacussu* snakes. Dotted lines indicate a hypothetical correspondence between transcript and protein abundances.

This hypothesis was confirmed when we evaluated the correlation between levels of transcripts and protein abundance of the isoforms with the most distinct distribution in the venoms: BJSUSVMPIII002, most highly expressed in small snakes, BJSUPLA002, most highly expressed in larger snakes, and BJSUSVMPIII009, evenly distributed expression despite the snake size. The transcriptional level of these three isoforms is at similar ranges in small snakes (Figure 6). However, the transcriptional levels of PLA002 increased proportionally to the snake size, with a corresponding increase in the expression of the isoform in the venom, whereas the transcript and venom concentration of BJSUSVMPIII002 decreases substantially (Figure 6). The transcriptional activity of BJSUSVMPIII009 was maintained around 1000 TMM in all snakes, as well as the EUSC in the venom, except for one small snake (Figure 6).

Recently, the genome of the prairie rattlesnake (*Crotalus viridis*) has been published, and it showed that promoter sequences of toxin genes present several binding sites for the transcription factors (TFs) Grainyhead-like protein 1 (GRHL1) and Nuclear factor 1 (NFI) [42]. Thus, we searched for the expression of GRHL1 and three genes of the NFI (i.e., NFIA, NFIB, and NFIX) in the transcriptome of *B. jararacussu* samples to understand if these regulatory mechanisms may be controlling the expression profile observed in the toxin genes. We noticed that GRHL1 presented a similar expression profile in all individuals analyzed (Supplementary Figure S3), which indicates that this TF may be evenly regulating the expression of most toxins in the venom glands of *B. jararacussu*. The NFIA presents a similar expression profile, whereas the NFIB and NFIX present distinct expression profiles among individuals (Supplementary Figure S3). The NFIB has a higher expression in small snakes and a lower expression in larger snakes and showed a negative correlation with the individuals’ SVL (R² = 0.6821/*p* = 0.042/slope b = −50.16). Although NFIX is more highly expressed in larger individuals, the correlation was not statistically significant (R² = 0.51/*p* = 0.1/slope b = 65.20). In addition, after evaluating the expression values (transcripts per million reads, TPM) of the PIII-class SVMPs transcripts, the BJSUSVMPIII002 isoform showed a positive correlation with NFIB (R² = 0.84/*p* = 0.01/slope b = 0.3555). These data and the prediction performed by Schield et al. 2019 [42] indicate the TFs NFI may be acting to fine-tune the expression profile observed among small and large individuals. However, the analysis was performed in silico, and further functional experiments are necessary to confirm such interactions and regulatory mechanisms.

### 2.4. Compositional Variability Lead to Functional Differences in the Toxic Activities of B. jararacussu Venom

**Figure 7:**
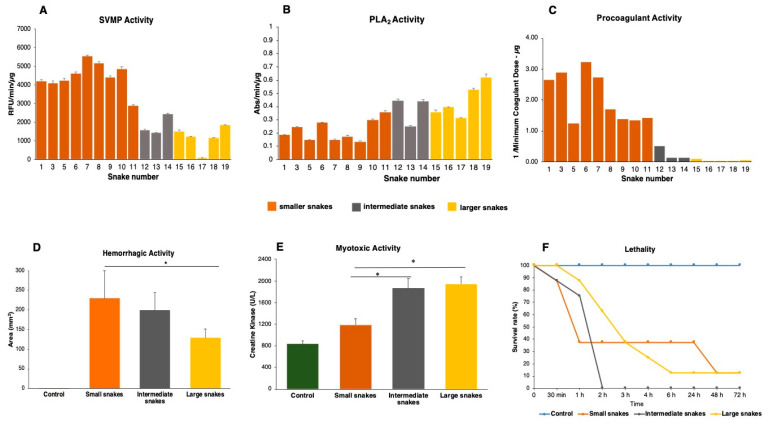
Functional activities of *Bothrops jararacussu* venom samples. (**A**)—Metalloproteinase activity (SVMP) was measured by fluorometric assays using the substrate Abz-AGLA-EDDnp. (**B**)—Phospholipase A_2_ (PLA_2_) activity was tested using the 4-nitro-3- [octanoyloxy] benzoic acid substrate. (**C**)—Procoagulant activity was evaluated by the amount of venom (µg) capable of inducing the coagulation of human citrated plasma in up to 60 s and is represented by the reciprocal Minimum Coagulant Dose (1/MCD). (**D**)—For hemorrhagic activity, 10 µg of each pool of venoms were injected in the dorsum of mice, and the hemorrhagic lesions were measured three hours after injection. (**E**)—The myotoxic activity was assayed using 50 µg of pools of venoms injected intramuscularly into the gastrocnemius muscle in Swiss mice, and after 3 h, the sera were assayed for creatine–kinase activity. (**F**)—For the evaluation of lethal activity, samples containing 200 μg of each venom in a final volume of 200 μL were injected intraperitoneally into groups of four mice. Phosphate-buffered saline (PBS)—was the control in all in vivo experiments. The results are representative of two independent experiments. * *p* < 0.05.

Considering that the major variability in venom composition was related to the abundance of the most important venom enzymes, we tested the functional activities of PLA_2_s and SVMPs and the impacts of their different expression levels on venom toxicity relative to snake size. All venom samples showed enzymatic activity on synthetic substrates for SVMPs and PLA_2_s (Figure 7A,B). As expected, the enzymatic activity of SVMPs showed a decrease as snake sizes increased, presenting a negative correlation with the size of the snakes (R^2^ = 0.749/*p* = 0.0001/slope b = −964.5) and a positive correlation with the abundance of the SVMPs in the venoms (R^2^ = 0.787/*p* < 0.0001/slope b = 3747). However, the enzymatic activity of PLA_2_s showed a different result: a weaker correlation with the total amount of PLA_2_s in the individual venoms (R^2^ = 0.248 /*p* = 0.041/ slope b = 850300) but still a positive correlation with the size of the individuals (R^2^ = 0.63/*p* = 0.0001/slope b = 0.00036). These results were not surprising, since the most abundant isoform (BJSUPLA002) is devoid of catalytic activity. In this case, the substrate hydrolysis was mostly caused by the acid isoforms, which are less abundant and equally distributed among the venoms, and due to the presence of other D-49 isoforms (BJSUPLA004 and 005), which are more expressed in the large specimens. 

In addition to activities on synthetic substrates, the toxic activities of the venoms were tested. The pro-coagulant activity was assessed as the Minimum Coagulant Dose (MDC), which is the amount of venom (µg) capable of inducing plasma coagulation in up to 60 s. The venoms of the smaller snakes (SVL < 603 mm) were more pro-coagulant with an MDC smaller than 1 µg. Individuals with an SVL between 600 and 770 mm had an intermediate MDC between 2 and 8 µg, and larger individuals (SVL > 900 mm) were less pro-coagulant with MDC ranging between 10 and 42 µg (Figure 7C).

To save the number of experimental animals, in vivo activities were evaluated using pools of venoms grouped according to size (SVL), the composition of the venom, and the in vitro activities as follows: pool 1 (P1), containing venom from smaller individuals (BJSU01–BJSU11, SVL 257–603 mm), pool 2 (P2), composed of venom from intermediate individuals (BJSU12–BJSU14, SVL 698–770 mm), and pool 3 (P3), containing venom from larger individuals (BJSU15–BJSU19, SVL 942–1230 mm). Animals injected with P1 had statistically significantly stronger hemorrhage than P3 (Figure 7D), and animals injected with P2 and P3 showed statistically significant higher myotoxic activity (Figure 7E). Animals injected with P1 showed no statistically significant difference in myotoxic activity from the PBS control. These results were in agreement with the fact that the venoms of smaller individuals have more PIII-class SVMPs and larger individuals have more myotoxic PLA_2_.

Despite the differences observed among the toxic activities of small intermediate and large snakes, our analysis on the lethality of venom pools in mice (Figure 7F) revealed that P1 and P3 pools induced mice death in comparable numbers and periods, with a survival rate of approximately 20% for both groups. However, P2 seems to be capable of inducing death faster, and within 2 h, all animals were dead. Although both SVMPs and PLA_2_s are present in moderate abundance in the venoms of snakes of intermediate sizes, the additive or synergistic action of these two groups of toxins could explain higher venom lethality.

## 3. Discussion

The toxic repertoire of *B. jararacussu* venom has long been studied, initially by isolation and sequence characterization of its major toxins and more recently by cDNA cloning and sequencing of individual components. Using protein chemistry approaches, Bothropstoxin1, Bothropstoxin2, and BthA-1 were characterized [33,35,43], and consisted, respectively, of prototypes of K49 basic, D49 basic, and D49 acidic PLA_2_s of venoms from *Bothrops* snakes. In addition to PLA_2_ analogues, other toxins have been isolated and characterized from *B. jararacussu* venom, such as BjcuL, a pro-inflammatory CTL [44,45], and Jararacussin, a thrombin-like SVSP [46]. Due to their low abundance in the venom, the primary structure of *B. jararacussu* proteinases have been reported mostly from cDNA cloning and the characterization of sequences generated by a directional cDNA library comprising 549 expressed sequence tags (ESTs) from the transcriptome of *B. jararacussu* venom glands [47]. Using this approach, it was possible to characterize the complete sequences of an SVSP called BjussuSPI [48], one PI-class SVMP called BjussuMPII [49], and one hemorrhagic PIII-class SVMP called BjussuMP-1P [41]. In this regard, our data show a greater capability to completely characterize the toxin arsenal of *B. jararacussu* venom. The complete sequences of 125 toxin isoforms were characterized from transcribed genes in *B. jararacussu* venom glands, from which 73 were detected in the venom proteome of at least one snake. Most of the sequences described previously were detected in our dataset.

Isoforms from different protein groups presented the highest similarity with toxins previously characterized from venoms of phylogenetically related species such as *Bothrops moojeni* (Supplementary Table S3). However, PII- and PIII-class SVMP isoforms are more dissimilar and presented identities with isoforms characterized in venoms from *Bothrops neuwiedi, Bothrops asper,* or with precursors characterized in transcriptomes of distinct species from *Crotalus*, *Agkistrodon*, *Gloydius*, *Sistrurus,* or *Protobothops* snakes, suggesting that they have not yet been described in *Bothrops* snakes. Some PIII-class isoforms clustered together with HF3 or Berythractivase isoforms that are present in minor proportions on *B. jararaca* and *B. erythromelas* venoms, respectively. In contrast, we did not detect any isoform presenting identity to Batroxhagin [50] or Jararhagin [51], which are considered “core toxins”, and these are abundant in venoms of different species of *Bothrops* and involved in the major toxic activities of the respective venoms [7]. With these data, we demonstrate that *B. jararacussu* venom has a high diversity of PIII-class metalloproteinases, and new components that have yet to be functionally characterized. In this regard, it is likely that *B. jararacussu* snakes express different gene clusters for venom metalloproteinases than the ones used by other species of *Bothrops*.

Concerning venom variability, it has already been observed that venoms from certain species of *Bothrops* vary throughout the species distribution [14,15,52]. However, the venom extracted from *B. jararacussu* collected from different regions of the species distribution (Southeastern and Southern Brazil) showed no distinguishable differences in the quantity of the main toxin families (Figure 2B). In contrast, high levels of variability in venom composition were observed according to the size of the snakes. Ontogenetic variability has already been shown in the venoms of *B. atrox* (from Colombia and Venezuela) and *B. asper,* in which a predominance of PIII-class SVMPs was observed in venoms from juvenile individuals, while PI-class SVMPs and PLA_2_s predominated the venoms from adult snakes [11,14,53]. The ontogenetic shift between PIII-class and PI-class SVMPs was also observed in *B. jararaca* venom [54]. Interestingly, in our study, PI-class SVMPs were not correlated with the increase in body size of the snakes, and venom variability was related mostly to PIII-class SVMPs in small snakes changing to basic PLA_2_s in larger ones. The same was observed in a recent study on ontogenetic variation in *B. jararacussu* venom [26] that also correlated the presence of PIII-class SVMPs in venoms of juveniles changing to PLA_2_s in adult snakes. 

These changes in venom composition have been mostly correlated to the sexual maturation of the snakes. However, most of these studies used a fixed threshold value of SVL as a measure to determine if snakes are in the juvenile or adult stages [17,26,30,55,56,57]. As shown here, this definition does not apply to *B. jararacussu*. For example, the study on ontogenetic variation in *B. jararacussu* venom performed previously [26] considered adult males with SVL ranging from 830 to 990 mm and females from 930 to 1200 mm SVL. Using histological analysis to characterize the maturation of the sexual organs, we were able to characterize individuals with SVL ranging from 500 to 700 as adults, which would normally be associated with juveniles according to their sizes. On the other hand, we were also able to detect individuals with SVL = 942 mm that were still juveniles and not yet sexually mature. The same result has already been reported for *B. atrox* and *B. jararacussu* snakes, in which small snakes may already be sexually mature, while larger ones may still be juveniles [22,58]. In this sense, analyzing the SVL parameter to infer the reproductive status may lead to erroneous conclusions. Thus, the most important thing is to always analyze snakes within the same size range and/or do a histological study to define the reproductive status.

Using histological characterization of the reproductive stage in this study, we ruled out that *B. jararacussu* venom composition changed according to the maturation of the snake reproductive stage. Instead, the replacement from PIII-class SVMPs to basic PLA_2_s occurred gradually, with a continuous correlation to snake growth during their entire life. However, there are still some unclear points on the eventual sex-related variability in venoms of adult *B. jararacussu* snakes. Aguiar and co-workers [26] reported the compositional and functional differences between venoms from males and females in juvenile and adult stages. Here, we detected significant differences between PIII-class SVMPs and PLA_2_s in venoms from males and females, but this difference was attributed to the larger size of the female snakes included in this study. The adult males captured for this study were smaller than females (SVL from 510 to 698 in males and 1086 to 1230 in females) and had their venoms included in the group of small snakes. Interestingly, the largest male included in this study (BJSU014) was 770 cm SVL long but classified as a sexually immature juvenile. The venom of this specimen was considered of intermediate composition, maintaining the relative proportions and isoforms of PIII-class SVMPs and PLA_2_ myotoxins. It is reasonable to suggest that the changes in venom composition are more frequent in females as they reach much larger sizes than males. *B. jararacussu* is a snake species with one of the highest sexual size dimorphism indices (SSD), which includes large female body size, a large litter, and large offspring size [22]. However, this is still unclear, and studies with higher numbers of large snakes should be carried out to solve this issue. 

The ontogenetic change in venom composition has already generated numerous discussions and is often associated with the levels of sex hormones and sexual maturation of snakes. Schonour et al. (2020) [59] showed that testosterone concentrations were correlated with the change in the composition of the venom, but the relationship was not strong enough to suggest causality. Although we did not measure testosterone concentrations in this study, we ruled out the supposition that the ontogenetic venom variability could be related to the levels of reproductive hormones present in the different stages of the snake’s reproductive life. However, our results clearly show that the variability of SVMPIII and PLA_2_ observed in *B. jararacussu* linearly correlated to the body size of snakes. This does not exclude the possibility that the levels of hormones directly linked to body growth such as somatotrophin, thyroid gland stimulant (TSH), and thyroid hormones (T3 and T4), which play an important role in growth, development, and metabolism, are influencing the expression of specific toxins according to the snake’s growth. Moreover, the possible mechanisms involved in snake venom ontogenetic variability are still unclear, and postgenomics may have a major influence on modifications in the composition of snake venoms during their life cycles. Well-supported evidence indicates that microRNAs may influence the ontogenetic pattern of venom protein translation in rattlesnakes, controlling the expression of SVMPs and PLA_2_s in venoms from adult or juvenile *Crotalus simus*, *C. tzabcan*, and *C. culminatus* snakes [60,61]. In other studies, an increased expression of translation initiation factors was observed and correlated to the transition of venom phenotype in *Bothriechis* species [62]. Translation initiation factors enhance translation by stabilizing mRNA and ribosomal complexes and may act as a translational regulatory mechanism [63], while microRNAs play important roles in regulating gene expression by interacting with the 3′ untranslated region of target mRNAs, inducing mRNA degradation and translational repression [64]. These mechanisms might be controlling, to some degree, the translation of venom toxin coding genes. Interestingly, we did not detect any putative regulatory elements acting on these regions of the toxin transcripts (data not shown). However, we observed the upregulation of PLA_2_ genes in adults and SVMP genes in small snakes in venom gland transcriptomes, demonstrating that post-transcriptional mechanisms did not contribute significantly to the phenotypic alteration during snake life cycles and suggest a more prominent and direct role for transcriptional regulatory events. In this aspect, GRHL1 showed a high and uniform expression in the venom glands of all snakes, while the NFIB presented a higher expression in small snakes. NFIB showed a negative correlation with the individuals’ SVL and a positive correlation with TPM values of the BJSUSVMPIII002 isoform. These data and the prediction performed by Schield et al. 2019 [42] indicate the TFs NFI may be acting to fine-tune the expression profile observed among small and large individuals. Another interesting aspect observed in this study was that the differential expression during ontogeny appeared to be on a locus-specific level rather than a protein-family level. The best example of that was the expression levels of major PIII-class SVMP isoforms in *B. jararacussu* snakes. During the ontogeny of the snakes, seven genes were down-regulated, three maintained their expression levels, and one was upregulated in adults relative to juveniles. Unfortunately, transcriptional regulatory events will be clarified only after the genome of *B. jararacussu* snake becomes available.

The size-related variability in the venom composition of *B. jararacussu* snakes may certainly be reflected in venom toxicity and is probably related to ecological and behavioral constraints observed during the snake’s life cycle. Several studies have shown experimentally that in different species of viperid snakes, the venoms of juvenile individuals are more pro-coagulant than venoms from adults [57,65,66,67]. The higher pro-coagulant activity of venoms from small snakes was also reported in human cases of snakebites [68,69]. The rapid blood clotting is related to a consumption coagulopathy characterized by the conversion of fibrinogen to fibrin and activation of coagulation factors, as well as interference in platelet function and fibrinolysis [70,71]. The ability to activate components of the coagulation cascade, such as factor X and II, is a characteristic of several pro-coagulant SVMPIIIs [72]. The higher coagulant activity of venom from smaller specimens observed in this study is in agreement with these previous observations and may be attributed to the differential expression of the metalloproteinase isoforms involved in the activation of prothrombin and factor X, probably BJSUSVMPIII002, which was more abundant in all smaller individuals and is structurally similar to Berythractivase, a calcium-independent activator of prothrombin [40]. Parallel to intense pro-coagulant activity, *B. jararacussu* venoms from smaller specimens were more hemorrhagic, whereas those from large individuals induce stronger myonecrosis. These data are in agreement with previous reports on the ontogenetic functional variability of *B. asper* venom [73]. However, in this study, we did not observe marked differences between the lethality of venoms from small or large snakes to rodents. In an elegant study on the effects of *B. asper* venom in rodents [74], the authors suggested that lethality was mostly related to peritoneal hemorrhage, increased vascular permeability, and coagulopathy, with a predominant role of SVMPs. Comparing this study to our results, it was reasonable to expect that the venoms of the smaller snakes, with more SVMPs in their composition, were more procoagulant and would be more lethal. However, our results show that the venom of small and larger snakes had comparable lethality, while the venom from intermediate individuals induces a faster death in mice. One explanation for such findings is that although both SVMPs and PLA_2_s are present in moderate amounts in the venoms of snakes of intermediate sizes, the additive or synergistic action of these two groups of toxins would play in favor of higher venom lethality.

One important aspect to analyze when comparing the toxicity of venoms from small and large snakes is the differences in diet and prey habits. In *B. jararacussu* species, juvenile snakes feed predominantly on ectothermic prey, such as amphibians, while large adult snakes feed mainly on mammals of varying sizes [24]. In addition, the primary role of venom in juveniles would correlate to prey immobilization, while in adults, the digestive role would increase as diet shifts to larger and heavier prey [75]. The behavior of amphibian capture by juveniles of *B. jararacussu* usually involves caudal luring, and after the bite, the snake retains the amphibian in the mouth until the amphibian ceases its movements and the ingestion process is started [76,77]. It has been observed that juveniles can press the prey against the substrate using the middle portion of the body to help immobilize the frog [77]. Thus, the venom of small snakes would be helping to kill the prey. For this purpose, the venoms from smaller snakes were selected to be more pro-coagulant and as previously shown, endogenous thrombin formation can induce a circulatory shock, which results in prey incapacitation [78]. Larger individuals feeding mostly on rodents have a different prey strategy. Rodents pose a risk to snakes, and snakes release rodents after the bite. Thus, the venom must act quickly to prevent the prey from escaping [76]. However, larger individuals have a less diverse venom, with high concentrations of myotoxic PLA_2_s and lower SVMP diversity, but this composition is apparently more efficient in the rapid immobilization of mammals, preventing them from moving away. Mackessy (2010) [79] suggests that the general occurrence of myotoxins in rattlesnakes’ venoms may facilitate immobilization in the absence of neurotoxic PLA_2_. Moreover, the amount of venom injected by larger snakes may be higher than by smaller snakes [80]. Thus, injecting more venom into the prey would compensate for the lower diversity of the venom by increasing the role of myotoxins in the immobilization/death of the prey.

## 4. Conclusions

We present here a comprehensive study on *Bothrops jararacussu* snake venom composition based on data obtained from 19 individuals of different sex, size, reproductive status, or geographical location. We carried out the first next-generation sequencing transcriptome (NGS) of venom glands from six individuals, which allowed a label-free characterization and quantification of venom toxins at the isoform level and evidencing the great diversity of PIII-class SVMPs in this venom. *B. jararacussu* venom shows up-regulation in the expression of myotoxic PLA_2_s and down-regulation of PIII-class SVMPs paralogues along the snake lifecycle, which is proportional to the snake size and not related to reproductive stage or geographical location. Accordingly, the hemorrhagic and procoagulant activities predominated in venoms of small snakes, while in large snakes, venoms were more myotoxic. Venom variability was modulated mostly at transcriptional levels of a limited number of paralogues. As result, coagulant and hemorrhagic venoms from small snakes might be related to predatory function, while the venom from large snakes seems more related to immobilizing by high tissue-damaging myotoxic activity.

## 5. Materials and Methods

### 5.1. Snakes and Venoms

Nineteen specimens of *B. jararacussu* were collected between 2017 and 2019 in the States of Rio de Janeiro, Santa Catarina, and São Paulo, Brazil (Supplementary Figure S1), under ICMBio/SISBIO permits 56576, 57585, and 66597; and INEA permits 025/2018. Snout-vent length (SVL) and tail length (TL) were measured for all individuals. The reproductive status of each snake was determined by macroscopic and histological morphological analyses as described [81]. Macroscopic measurements were taken of the diameter of the primary and secondary follicles for females and the length and width of the testicles for males. For the characterization of sexual maturity, females were considered mature when they presented any of the following characteristics: (1) secondary follicles, (2) corpus luteum or albicans, (3) pregnancy, or (4) distended oviduct. For males, the confirmation of sexual maturity was determined by the presence of folded deferent ducts with the presence of sperm, testicles with sperm, and hypertrophied renal sexual segment.

Venom samples were collected using manual extraction techniques soon after snake capture, without any previous feeding at the lab. Venom samples were individually freeze-dried and kept at −80 °C until used for proteomics and functional tests. Four days after venom extraction, venom glands were dissected and immediately transferred to RNALater (Invitrogen, Life Technologies Corp., Waltham, MA, USA) and stored at −80 °C until RNA extraction and mRNA isolation.

### 5.2. Transcriptome 

#### 5.2.1. RNA Extraction and cDNA Library Construction and Sequencing

The RNA was extracted based on the use of guanidine isothiocyanate followed by phenolic extraction as previously described by Chomczynski and Sacchi [82], with slight modifications. Initially, the total RNA was extracted with Trizol and quantified by Quant-iT RiboGreen RNA reagent and Kit (Invitrogen, Life Technologies Corp., Waltham, MA, USA). Then, quality check control of the integrity of extracted RNA was performed in an Agilent 2100 Bioanalyzer (Santa Clara, Ca, USA) using an Agilent RNA 6000 Nano kit. Libraries were prepared for each sample. One μg of total RNA was used with Illumina TruSeq Stranded RNA HT kit (Hayward, CA, USA) consisting of TruSeq Stranded RNA HT/cDNA Synthesis PCR, TruSeq Stranded RNA HT/Adapter Plate Box, and TruSeq Stranded HT mRNA. Libraries were validated by considering their fragment size distribution and their quantification. Then, the quantification of each library was performed by Real-Time PCR using the KAPA SYBR FAST Universal qPCR kit (Merck, Darmstadt, Hesse, Germany), according to the manufacturer’s protocol, using the StepOnePlus™ Real-Time PCR System (Waltham, MA, USA). Aliquots of each cDNA library were diluted to a concentration of 2 nM. Next, a pool of all samples with 5 μL of each library was prepared, and the concentration of the pool was again determined by Real-Time PCR. The cDNA libraries were sequenced on the Illumina HiSeq 1500 System (Hayward, CA, USA) into a Rapid paired-end flowcell in 300 cycles of 2 × 150bp paired-end strategy.

#### 5.2.2. Transcriptome Assembly

Initially, the reads generated were checked for cross-contamination among samples sequenced in the same flow cell by using an in-house Python script as previously described by Hofmann et al. [83]. Then, the adapter sequences and low-quality reads were trimmed using TrimGalore [84]. The reads were merged using the PEAR software [85]. The merged and unmerged reads were grouped and used as input for de novo transcriptome assembly. Three assemblers were used to maximize the chances of obtaining full-length toxin transcripts following [31]: Trinity (v2.4.0; [86], SeqMan NGen v. 14 (DNAStar, Inc., Madison, WI, USA), and an in-house assembler named “Extender” [87]. Trinity and SeqMan NGen were used with their default settings, whereas Extender was applied setting two minimum overlaps for extension (120 bp and 150 bp) and the minimum quality score of at least 20 at all base positions for a read to be considered for extension. 

#### 5.2.3. Transcriptome Annotation (Toxins)

The contigs from each individual were aligned against the Toxicofera/Uniprot protein databases using the BLASTx [88]. The result was filtered considering 90% as the limit of the minimum percentage of the total length of a match to exclude small fragments, exons, etc. Then, the probable open reading frames (ORFs) of each contig were visualized using SeqBuilder Pro (DNAStar, Inc., Madison, WI, USA), and the most probable coding regions according to the alignment result were selected and manually annotated. Chimeras and duplicates were removed both by manually examining the read mapping profile and by using the ChimeraCheck software [83]. The coding sequences annotated from all individuals were grouped into a single file at 98% identity using the cd-hit-est [89,90] and resulted in a final master transcriptome set for *B. jararacussu.* The expression of each contig was estimated using the RSEM software [89] after mapping reads from each sample using bowtie2, and measured in transcripts per million reads (TPM) [91]. Then, we performed an additional TMM (trimmed mean of M-values) scaling normalization that aimed to account for differences across all samples by using the EdgeR package [92] in the R environment. Raw transcriptomic data are available at NCBI’s GenBank under Bioproject accession number PRJNA659835 and Biosamples accession number at SRX9057906–SRX9057911. Curated sequences (CDS and translated proteins) generated in this work are available in the Supplementary Table S3.

In addition, we searched for putative regulatory elements that may be acting in the transcriptional and post-transcriptional levels of the transcripts. First, to detect the transcriptional factors (TFs) GRHL1 and NFI in the transcriptome assembly, we performed a coding sequence prediction using CodAn (v1.0; [93]) with the vertebrate full model. Next, we performed a BLAST search using a database containing the GRHL1 and NFI peptide sequences available from Swiss-Prot, Ensembl, and previously published snake transcriptome assemblies from the TSA database [9,87,94,95]. Then, we performed the quantification using RSEM as previously described.

### 5.3. Venom Proteome

The proteomic characterization was performed by reversed-phase nanochromatography coupled to nanoelectrospray high-resolution mass spectrometry. Initially, replicates of each venom sample (50 µg of protein) were denatured by urea, reduced by triethylphosphine, and alkylated by iodoethanol before treatment with trypsin solution (0.2 µg/µL). The peptide concentration of each sample was quantified with the Qubit 2.0 Fluorometer (Invitrogen, Life Technologies Corp., Waltham, MA, USA). Peptide desalting was performed using reverse-phase Empore C18-SD columns, 4 mm/1 mL (Sigma-Aldrich, Darmstadt, Hesse, Germany). The C18 column was equilibrated by 100% acetonitrile (ACN) and washed by 0.1% *v*/*v* trifluoroacetic acid (TFA) (in water) twice. The peptide pellet was resuspended in 0.1% *v*/*v* TFA and bound onto the column. The C18 column was washed by 0.1% *v*/*v* TFA twice, and peptides were eluted by a solution containing 50% *v*/*v* ACN, 0.1% *v*/*v* TFA, and 70% *v*/*v* ACN, 0.1% TFA. The eluate was dried in a speed-vacuum. The Nano LC–MS/MS analysis was performed in duplicate. Peptide samples were resuspended in 0.1% TFA and analyzed using an EASY-nLC system (Thermo Scientific, Waltham, MA, USA) coupled to an LTQ-Orbitrap Velos mass spectrometer (Thermo Scientific, Waltham, MA, USA). The peptides were loaded onto a C18 PicoFrit column (C18 PepMap, 75 µm id × 10 cm, 3.5 µm particle size, 100 Å pore size; New Objective, Ringoes, NJ, USA) and separated with a gradient from 100% mobile phase A (0.1% TFA) to 34% phase B (0.1% TFA, 95% ACN) during 60 min, 34–95% in 15 min, and 5 min at 95% phase B at a constant flow rate of 250 nL/min. The LTQ-Orbitrap Velos was operated in positive ion mode with data-dependent acquisition. The full scan was obtained in the Orbitrap with an automatic gain control (AGC) target value of 10e6 ions and a maximum fill time of 500 ms. Precursor ion scans were acquired at a resolution of 60,000 FWHM (Full Width of the peak at Half its Maximum) in the 400–1500 m/z mass range. Peptide ions were fragmented by collision induced dissociation-CID MS/MS using a normalized collision energy of 35. The 20 most abundant peptides were selected for MS/MS and dynamically excluded for 30s. All raw data were processed and searched against an in-house database using the search tools Mascot (Matrix Science, London, UK; version 2.4.1). The database used to identify the MS/MS spectra is composed of the full-length precursor proteins derived from the combination of *B. jararacussu* transcriptomes described in this manuscript. We validated the MS/MS-based peptide and protein identifications by using Scaffold (v4.9.0; Proteome Software Inc., Portland, OR, USA). Peptide identifications were accepted if they could be established at >99.0% probability by the Scaffold local false discovery rate-FDR algorithm. Peptide identifications were also required to exceed specific database search engine thresholds, such as Mascot ion scores >40.0 and/or X! Tandem –Log (E-value) scores >2.0. Protein identifications were accepted if they could be established at >99.0% probability to achieve an FDR <1.0%. Protein probabilities were assigned by the Protein Prophet algorithm [96]. The quantification of proteins related to the general abundance in each venom was based on normalized Total Spectra Counts, and for identifications at the isoform level, venom analyses were expressed as normalized Exclusive Unique Spectral Counts, corresponding to the number of spectra attributed to proteotypic peptides of a given protein entry present in the database. 

### 5.4. Sequence Alignments and Gene Tree Analyses

The prediction of biological function for *B. jararacussu* sequences was inferred using the phylogenetic analysis to identify homologous toxins in other snake species with functional activities already described. The sequence alignments and gene tree inference were performed only for highly expressed toxins in the proteome. Putative mature protein sequences (Supplementary Table S2) were subjected to Blastp search (ncbi.nlm.nih.gov) to find the closest primary-structure-related toxins, which had their function experimentally characterized. The sequences were aligned with the iterative refinement method E-INS-i using a BLOSUM62 scoring matrix as implemented in MAFFT (v7.450; [97]). The best-fit models of evolution were selected for each alignment using the corrected Akaike Information Criterion (AICc) implemented in ProtTest (v3.4.2; [98]). Maximum likelihood (ML) phylogenetic trees were estimated using RAxML (v8.2.12; [99]). The rapid bootstrap algorithm performed by RAxML (–fa) was used to generate 1000 pseudo replications of non-parametric bootstrap aiming to estimate support values (BS) for each clade. This algorithm also conducts a complete search of ML using each 5th bootstrap tree as a starting tree for the rapid hill-climbing search (total of 200 starting trees).

### 5.5. Functional Activities 

#### 5.5.1. Enzymatic Assays on Synthetic Substrates

SVMPs and PLA_2_ enzymatic activities were assayed using synthetic substrates according to the procedures previously standardized in our lab [100]. For SVMPs, venom samples (10 µg) were incubated with 50 μM of FRET (Fluorescence Resonance Energy Transfer) Abz-AGLA-EDDnp substrate (Peptide International), and the enzymatic reactions were monitored in a SpectraMax^®^ M2 fluorimeter (Molecular Devices, San Jose, CA, USA) with excitation at 340 nm and emission at 415 nm, at 37 °C in kinetic mode over 10 min with a read range of 1 min. The results were expressed in Relative Fluorescence Units-RFU/min/µg. The PLA_2_ activity of venom samples (20 µg) was assayed using 500 μM of the substrate 4-nitro-3-[octanoyloxy] benzoic acid (Enzo Life Sciences, New York, NY, USA) incubated for 40 min at 37 °C and activity determined according to the absorbance at 425 nm and expressed as Absorbance/min/μg of venom. 

#### 5.5.2. Coagulant Activity

The coagulant activity of individual venoms was assessed in citrated human plasma from healthy volunteers under the approval of the Ethical Committee (CAAE: 89499218.8.0000.5377). Participants read and signed the written informed consent form before enrollment. Different concentrations of venom samples were diluted to in 25 μL of phosphate-buffered saline (PBS), incubated with 100 μL of plasma at 37 °C, and the clotting time measured in a coagulometer (Diagnostica Stago, START 4, Parsippany, NJ, USA). The coagulant activity was expressed as Minimum Coagulant Dose (MCD), which corresponds to the amount of venom that induces clotting of human plasma in 60 s.

#### 5.5.3. In Vivo Venom Activities

The amount of venom extracted from each snake was not sufficient to perform in vivo experiments individually. Thus, the venoms were grouped in 3 pools according to snake size: Pool 1 comprised venoms from smaller individuals (BJSU1–BJSU11, SVL 257–603 mm), pool 2 included a group of intermediate individuals (BJSU12–BJSU14, SVL 698–770 mm), and pool 3 grouped venoms of larger individuals (BJSU15–BJSU19, SVL 942–1230 mm). Hemorrhagic, myotoxic, and lethal activities were carried out using male Swiss mice (18–20 g) kept under controlled temperature and light periods with water and feed ad libitum, under the approval of the Butantan Institute Ethics Committee on Animal Use (Protocol Number: 5091020819). Briefly, for hemorrhagic activity, samples containing 10 µg of each venom pool, diluted in 50 µL of PBS, were injected intradermally into the dorsal skin of mice. After 3 h, the animals were euthanized in a CO_2_ chamber, the dorsal skin was removed, and the hemorrhagic halos were measured. Groups of 3 animals were tested, and control group animals were injected with PBS only. The myotoxic activity was assayed using 50 µg of venom pools in 30 µL of PBS, which was injected intramuscularly into the gastrocnemius muscle in Swiss mice. After 3 h, the animals were bled via ophthalmic plexus, and the sera were assayed for creatine–kinase activity with a commercial kit CK-UV (Bioclin, Belo Horizonte, MG, Brazil), according to the manufacturer’s instructions. Groups of 4 animals were tested, and animals from the control groups were injected with PBS only. To determine the lethal activity as described by Moretto Del-Rei et al. [16], samples containing 200 μg of each venom pool, in a final volume of 200 μL of PBS, were injected intraperitoneally into groups of 4 mice. The animals were monitored hourly until the sixth hour, and then at 12, 24, and 48 h after the injection of the samples. The times of death for each group were recorded in each time interval. Subsequently, the data obtained were graphed on a survival curve plot. All the experiments of functional activities were repeated at least 2 times, and the results represent the mean ± SE of the results obtained.

### 5.6. Statistical Analysis

The statistical analysis was performed using the software GraphPad Prism (GraphPad Software, Inc., San Diego, CA, USA). The normality of the data was assessed using the D’Agostino–Pearson normality test and normal probability plots. Data were log-transformed when necessary to meet the assumptions of parametric statistical tests. We used a correlation analysis (Pearson and Spearman correlation) and multiple linear regression to verify the association between the SVL, sex, locality of collection, reproductive status, and expression data of the transcripts or/and enzymatic activities. Differences between two groups were analyzed by the Student’s *t*-test (Unpaired or Nonparametric test, assuming normal Gaussian distributions) or One-Way ANOVA using the software GraphPad Prism (GraphPad Software, Inc., San Diego, CA, USA). Differences between groups were considered statistically significant at * *p* < 0.05; ** *p* < 0.01 and *** *p* < 0.001.

## Supplementary Materials

The following are available online at https://www.mdpi.com/2072-6651/12/12/791/s1, Figure S1: Geographical distribution of the nineteen *Bothrops jararacussu* snakes collected between 2017 and 2019 in the States of Rio de Janeiro, Santa Catarina and São Paulo, Brazil; Figure S2: Distribution of the predicted toxin families expressed in the venom glands of 6 specimens of *Bothrops jararacussu* identified from transcriptomic analysis; Figure S3: Distribution of the predicted Transcriptional Factors (TFs) expressed in the venom glands of 6 specimens from *Bothrops jararacussu;* Table S1: Information on all *Bothrops jararacussu* snakes used in this study collected in Brazil between 2017 and 2019 in different seasons; Table S2: Number of reads obtained after sequencing by Illumina of the mRNA present in the *Bothrops jararacussu* venom gland and the number of contigs generated after *De novo* assembly; Table S3: Identification and annotation of the toxin sequences from *B. jararacussu* venom gland transcriptomes; Table S4: Expression levels of independent isoforms in the venom of individual *Bothrops jararacussu* snakes; Table S5: Abundance of trancripts coding for the toxin isoforms in venom glands of individual *Bothrops jararacussu* snakes.
