# Supplementary material for: Size Matters: An Evaluation of the Molecular Basis of Ontogenetic Modifications in the Composition of Bothrops jararacussu Snake Venom
Source: Toxins (Basel). 2020 Dec 11;12(12):791. doi: 10.3390/toxins12120791 (PMC7763748; doi:10.3390/toxins12120791)
Supplement: Supplementary file 1 [file toxins-12-00791-s001.zip › toxins-988488 corrected supple/toxins-988488 supple for final.pdf]

Supplementary Materials: Size Matters: An Evaluation of the Molecular Basis of Ontogenetic Modifications in the Composition of *Bothrops jararacussu* Snake Venom

Luciana A. Freitas-de-Sousa, Pedro G. Nachtigall, José A. Portes-Junior, Matthew L. Holding, Gunnar S. Nystrom, Schyler A. Ellsworth, Noranathan C. Guimarães, Emilly Tioyama, Flora Ortiz, Bruno R. Silva, Tobias S. Kunz, Inácio L. M. Junqueira-de-Azevedo, Felipe G. Grazziotin, Darin R. Rokyta and Ana M. Moura-da-Silva

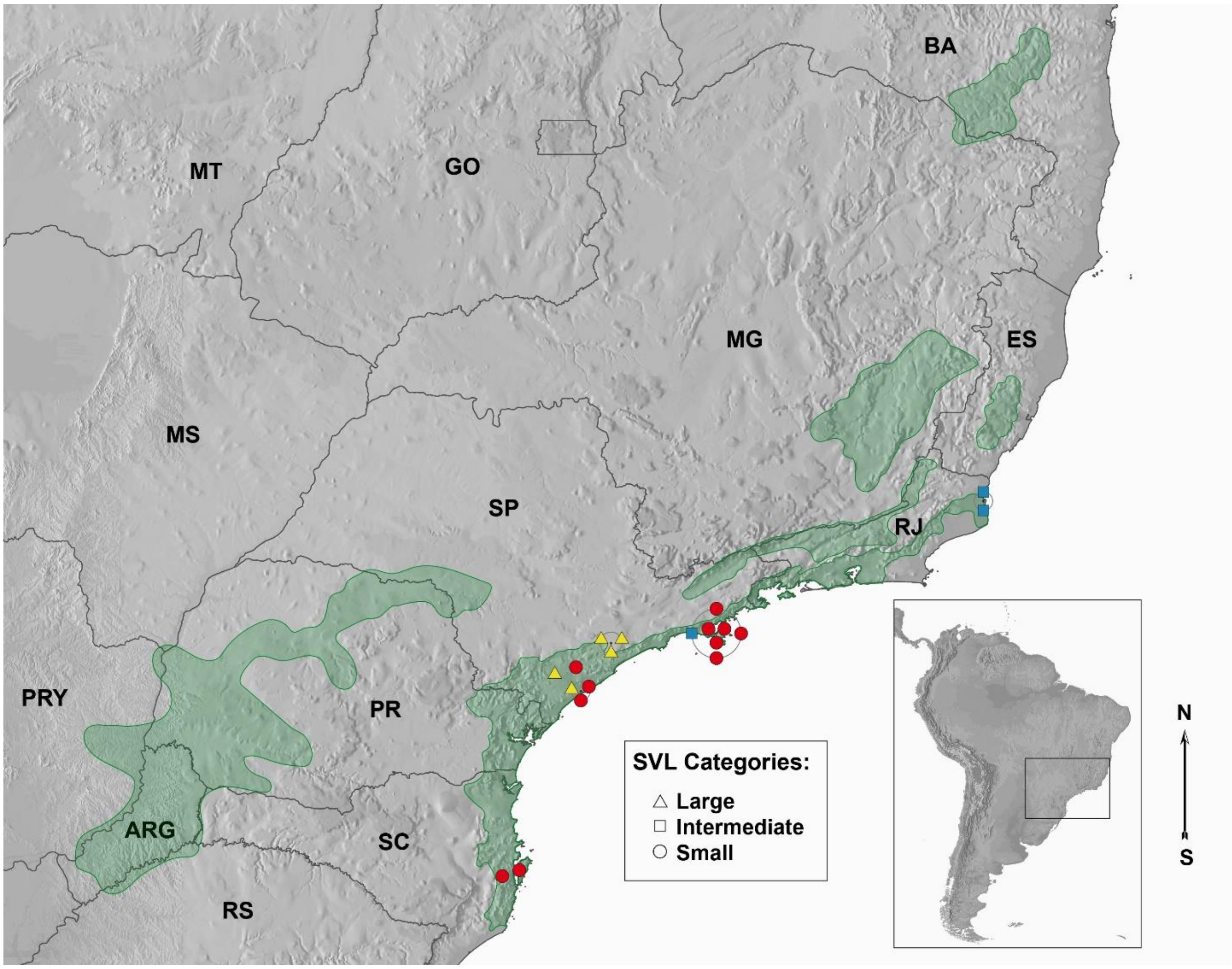

Figure S1. Geographical distribution of the nineteen *Bothrops jararacussu* snakes collected between 2017 and 2019 in the States of Rio de Janeiro, Santa Catarina and São Paulo, Brazil.

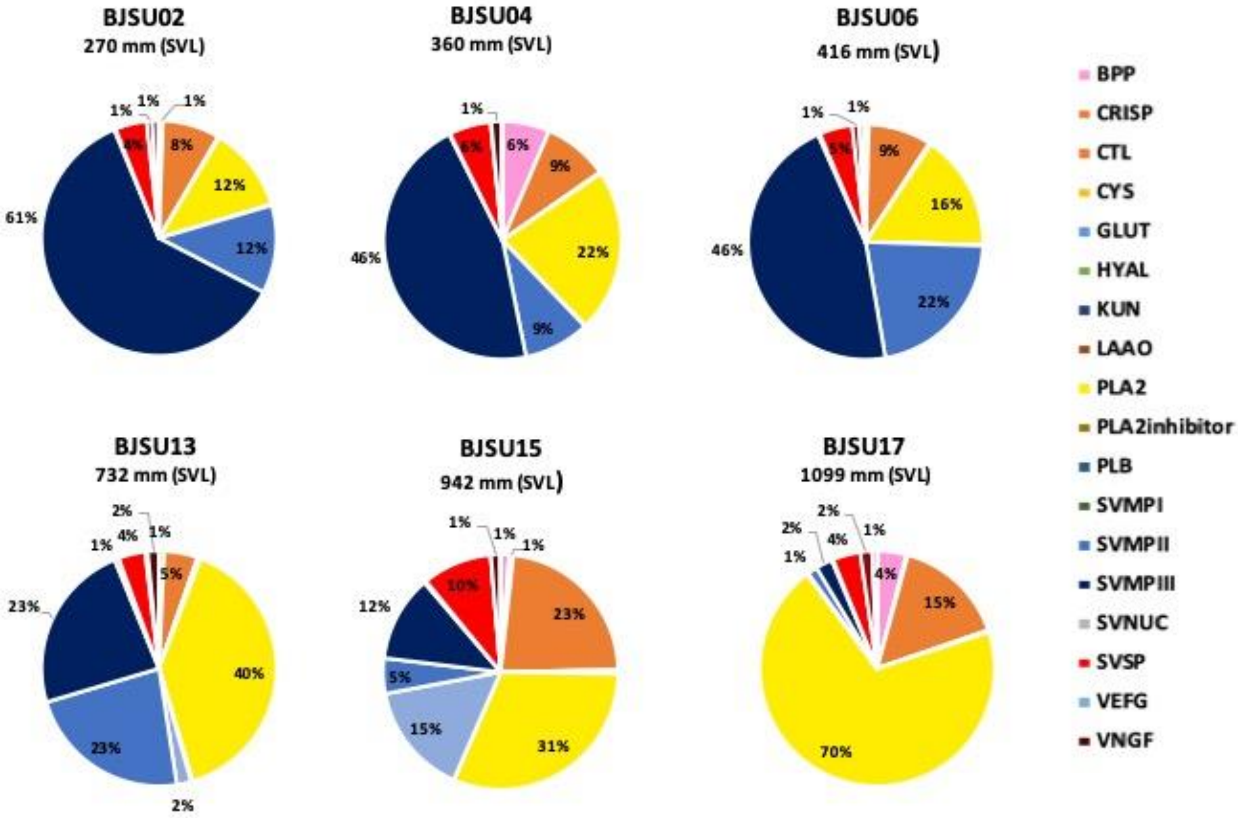

Figure S2. Distribution of the predicted toxin families expressed in the venom glands of 6 specimens of *Bothrops jararacussu* identified from transcriptomic analysis.

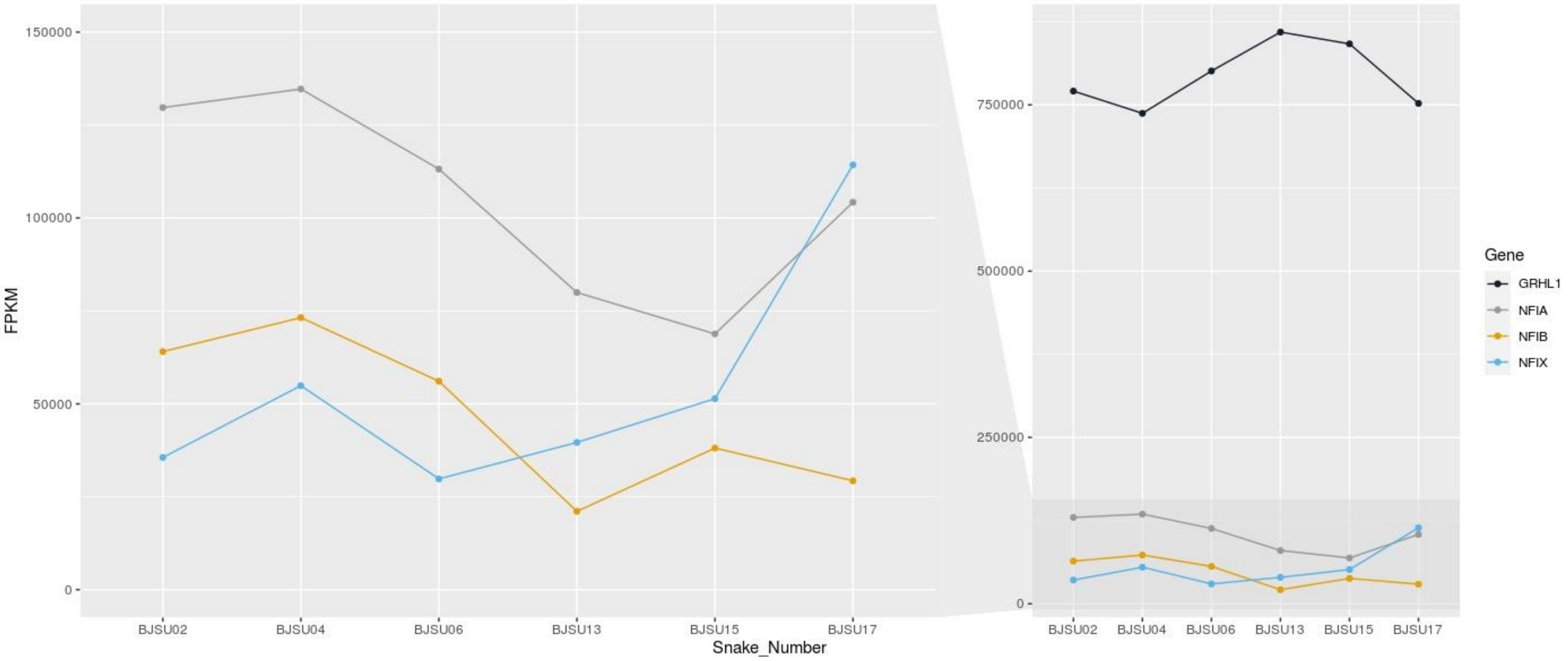

Figure S3. Distribution of the predicted Transcriptional Factors (TFs) expressed in the venom glands of 6 specimens from *Bothrops jararacussu*.

Table S1. Information on all *Bothrops jararacussu* snakes used in this study collected in Brazil between 2017 and 2019 in different seasons.

Snake Code	Sample Origin	Number Biota Project	Number IB Collection	SVL (mm)	TL (mm)	TotalL (mm)	Sex	Reproductive Status*	Use
BJSU01	Florianópolis-SC	SB0970	IBSP91234	257	45	302	M	juvenile	P
BJSU02	Iguape-SP	SB0335	IBSP89934	270	48	318	M	juvenile	T/P
BJSU03	Ilhabela-SP	SB0819	IBSP90672	350	50	400	F	juvenile	Prot
BJSU04	Juquiá-SP	SB0147	Nd	360	57	417	F	juvenile	T/P
BJSU05	Ilhabela-SP	SB0818	IBSP90671	364	63	427	M	juvenile	Prot
BJSU06	Iguape-SP	SB0334	IBSP89933	416	80	496	M	juvenile	T/P
BJSU07	São Sebastião-RJ	SB0054	Nd	445	56	501	F	juvenile	P
BJSU08	Ilhabela-SP	SB0822	IBSP90675	480	63	543	F	juvenile	P
BJSU09	Águas Mornas-SC	SB0803	IBSP90625	510	93	603	M	adult	P
BJSU10	Ilhabela-SP	SB0823	IBSP90676	530	95	625	M	adult	P
BJSU11	Ilhabela-SP	SB0820	IBSP90673	603	100	703	M	adult	P
BJSU12	Ilhabela-SP	SB0821	IBSP90674	698	135	833	M	adult	P
BJSU13	São João da Barra-RJ	SB0339	IBSP89940	732	100	832	F	juvenile	T/P
BJSU14	São João da Barra-RJ	SB0349	IBSP89945	770	Nd	Nd	M	juvenile	P
BJSU15	Juquitiba-SP	SB0394	IBSP90016	942	133	1075	F	juvenile	T/P
BJSU16	Sete Barras-SP	SB0100	MZUSP23111	1086	150	1236	F	adult	P
BJSU17	Juquitiba-SP	SB0097	MZUSP23108	1099	148	1247	F	adult	T/P
BJSU18	Iguape-SP	SB0324	IBSP90100	1120	Nd	Nd	F	adult	P
BJSU19	Juquitiba-SP	SB0096	MZUSP23109	1230	150	1380	F	adult	P

SVL-Snout-Vent Length; TL- Tail Length; TotalL- Total Length; * Reproductive Status defined by histological analyses; P – Proteomics; T- Transcriptomics; Nd- not determined.

Table S2. Number of reads obtained after sequencing by Illumina of the mRNA present in the *Bothrops jararacussu* venom gland and the number of contigs generated after *De novo* assembly.

Snake Code	Number of Reads Generated	% of Merged*	Number of Contigs Generated			
			Extender	Ngen	Trinity	Total
BJSU02	32,469,211	80.98	2,078	12,002	78,294	92,374
BJSU04	27,981,666	88.75	2,218	9,973	75,872	88,063
BJSU06	14,467,807	81.13	1,679	7,673	62,729	72,081
BJSU13	17,598,257	83.38	1,829	8,519	62,697	73,045
BJSU15	17,720,275	84.11	1,802	6,345	60,414	68,561
BJSU17	33,042,215	84.88	2,289	10,004	102,613	114,906

* The paired reads were merged based on their 3′ overlaps using PEAR version 0.9.6 (Zhang et al. 2014).
